# Advancements in the use of enemas for treating urinary calculi

**DOI:** 10.3389/fruro.2026.1737660

**Published:** 2026-03-13

**Authors:** Min Ling, Yujie Li, Liuyang Yang, Qinqin Song, Yu Sun, Chong Zhang, Yuhao Zhao, Shuaishuai Song, Li Xu, Yang Zhang, Hongbing Gu, Shengli Wang, Qilun Zhang, Yongfei Yang

**Affiliations:** 1 Department of Urology, The Third People’s Hospital of Bengbu, Bengbu, Anhui, China; 2 Core Research Facility, The Third People's Hospital of Bengbu Affiliated to Bengbu Medical University (Bengbu Central Hospital), Bengbu, China; 3 Department of Central Laboratory, The Third People’s Hospital of Bengbu, Bengbu, Anhui, China; 4 Department of Obstetrics and Gynecology, Bengbu First People’s Hospital, Bengbu, Anhui, China

**Keywords:** enema, integrative medicine, traditional Chinese medicine, urinary calculi, Western medicine

## Abstract

Urinary calculi are a prevalent condition within the urinary system, with treatment options ranging from traditional surgical and non-invasive methods to emerging alternative therapies. Recently, enema therapy utilizing traditional Chinese medicine (TCM) and Western medicine has gained attention as a non-conventional approach, offering unique advantages in managing urinary stones. Unlike conventional oral or surgical treatments, enema therapy enables direct colonic drug delivery, reduces systemic adverse effects, and may modulate gut microbiota and oxalate metabolism, thereby providing a novel mechanistic basis for stone prevention and expulsion. This paper provides a comprehensive review of the mechanisms, clinical applications, and safety considerations of TCM and Western medicine enema therapies. For the first time, we systematically integrate traditional theoretical frameworks with modern biomedical evidence, including microbiota-mediated oxalate degradation and detailed clinical operation parameters, to highlight the complementary and synergistic effects of combined TCM–Western medicine enemas. By analyzing both their individual and combined effects, this study aims to furnish clinicians with valuable insights to optimize treatment strategies and promote further development of enema therapy for urinary calculi.

## 1 Introduction

**Figure 1:**
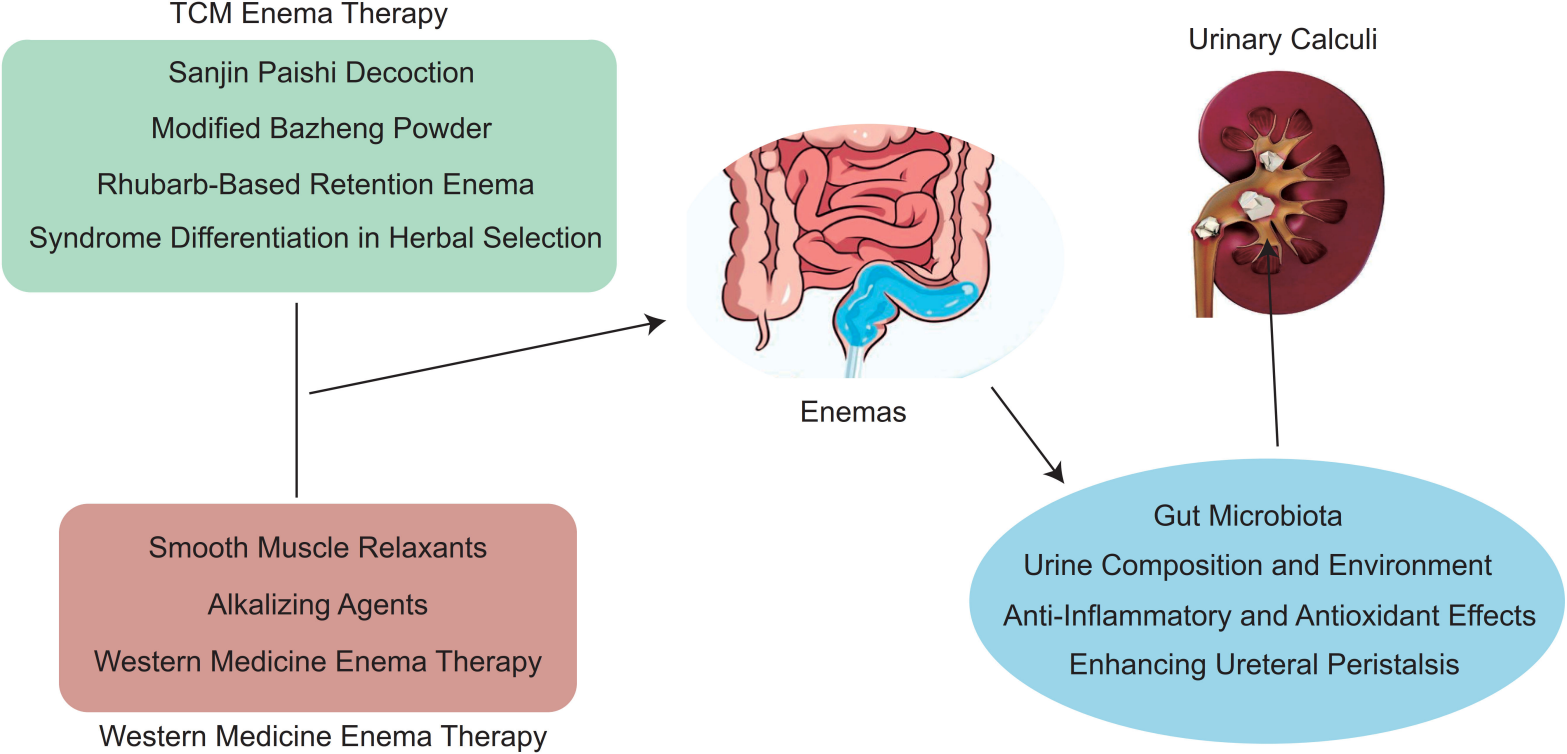
Mechanisms of TCM and Western medicine enema therapies in urinary calculi management.

The incidence of urinary calculi is increasing globally, posing a significant burden on healthcare systems and adversely affecting patients’ quality of life (1). This upward trend is driven by multiple factors, including dietary habits, sedentary lifestyles, metabolic disorders, and genetic predispositions, which collectively contribute to stone formation. Urinary stones not only cause acute symptoms such as renal colic, hematuria, and urinary tract obstruction, but they can also lead to chronic complications including recurrent infections, impaired renal function, and long-term morbidity. Consequently, urinary calculi represent a major public health concern that demands effective prevention and treatment strategies. Traditional treatment methods, such as surgical stone removal and extracorporeal shock wave lithotripsy (ESWL), have demonstrated efficacy but are associated with limitations, including invasiveness, postoperative complications, and high recurrence rates (2). Moreover, these conventional approaches often fail to address underlying metabolic or microbiota-related factors that contribute to stone formation, limiting long-term effectiveness. Herbal and natural therapies have also been widely applied in the management of urinary tract disorders, including stone prevention and symptom relief (3, 4). Recent studies suggest that certain herbal compounds can modulate urinary pH, reduce calcium oxalate supersaturation, and exhibit anti-inflammatory and antioxidant effects, thereby complementing conventional therapies. These challenges have led to the exploration of alternative approaches, including enema therapy with TCM and Western medicine, which offers unique advantages due to its localized delivery and novel mechanisms of action (**Figure 1**). Specifically, enema therapy enables direct absorption of active compounds through the colonic mucosa, enhances ureteral peristalsis, regulates urine composition, and may modulate gut microbiota, providing both preventive and therapeutic benefits. This method has garnered increasing attention as a promising adjunct or standalone therapy for urinary calculi, highlighting the potential of integrating traditional and modern medicine to improve clinical outcomes and reduce recurrence rates.

## 2 TCM enema therapy for urinary calculi

### 2.1 Selection of TCM formulas

#### 2.1.1 Sanjin Paishi decoction

Sanjin Paishi Decoction is a commonly used formula composed of Herba Lysimachiae (Jinqiancao), Endothelium Corneum Gigeriae Galli (Jineijin), and Lygodium japonicum (Haijinsha) (5). These herbs exhibit complementary effects: Jinqiancao inhibits calcium oxalate crystallization and aggregation while enhancing ureteral peristalsis, facilitating stone expulsion. Jineijin promotes digestion and strengthens ureteral contractions, aiding stone movement. Haijinsha relaxes ureteral smooth muscle, alleviating spasms and improving urinary flow (6).

#### 2.1.2 Modified bazheng powder

Modified Bazheng Powder is another frequently used formula. Key ingredients include Dianthus superbus (Qumai) and Polygonum aviculare (Bianxu), which promote ureteral peristalsis and enhance urinary flow. Other components, such as Talcum (Huashi) and Akebia (Mutong), clear heat and drain dampness, while Gardenia (Zhizi) and Rhubarb (Dahuang) reduce inflammation, improve blood circulation around stones, and facilitate stone loosening and expulsion (7).

#### 2.1.3 Rhubarb-based retention enema

Rhubarb (Dahuang) is widely used in retention enemas, often in combination with other herbal decoctions. Oral administration of rhubarb alone may yield limited results, but its use in enemas has demonstrated enhanced efficacy in promoting stone expulsion (8).

#### 2.1.4 Syndrome differentiation in herbal selection

For heat-dampness type urinary calculi, herbs such as Phellodendron (Huangbai) and Coix lacryma-jobi (Yiyiren) are commonly used to clear heat, drain dampness, and promote urination (9, 10). Phellodendron exhibits antibacterial and anti-inflammatory effects, which help alleviate urinary tract inflammation. Meanwhile, Coix seed promotes diuresis and dampness elimination, strengthens the spleen, and aids in the expulsion of urine, thereby reducing the concentration of substances that form stones in the urine. For qi stagnation and blood stasis types, Peach Kernel (Taoren) and Safflower (Honghua) are added to improve microcirculation, reduce adhesion around stones, and alleviate pain. Peach kernel improves microcirculation and reduces blood viscosity, ensuring smooth blood flow around the stone, minimizing adhesions, and facilitating stone expulsion. Safflower, on the other hand, enhances blood circulation, alleviates stasis, and relieves pain caused by stone obstruction.

### 2.2 Mechanisms of action

#### 2.2.1 Regulating urine composition and environment

Certain bioactive compounds in TCM can alter urine pH and ion concentrations, reducing the supersaturation of stone-forming substances such as calcium oxalate (11). For instance, certain alkaloid components in traditional Chinese medicine can bind with calcium ions in the urine, reducing the saturation of calcium oxalate and thereby decreasing the risk of stone formation. Additionally, traditional Chinese medicine can modulate the levels of substances like citrate in the urine. Citrate has the ability to chelate calcium, preventing the deposition of calcium salts and the formation of stones.

#### 2.2.2 Enhancing ureteral peristalsis

Active ingredients in TCM may act on smooth muscle cells in the ureter, modulating ion channels and neurotransmitters to enhance peristalsis and facilitate stone expulsion (12). For example, by increasing intracellular calcium ion concentration, traditional Chinese medicine can activate calmodulin-dependent protein kinases, which promote the contraction of ureteral smooth muscle, thereby facilitating the downward movement of stones. Moreover, traditional Chinese medicine may also regulate the release and action of neurotransmitters such as acetylcholine in the ureter, enhancing its peristaltic function.

#### 2.2.3 Anti-inflammatory and antioxidant effects

Urinary stones often induce inflammation and oxidative stress. Flavonoids and polyphenols in TCM exhibit significant anti-inflammatory and antioxidant effects, reducing cytokine release and oxidative damage, thereby aiding stone expulsion and urinary tract repair. They can inhibit the release of inflammatory cytokines such as interleukin-6 (IL-6) and tumor necrosis factor-alpha (TNF-α), thereby reducing inflammatory cell infiltration. Additionally, they help scavenge free radicals, protecting the urinary tract mucosal cells from oxidative damage. This not only facilitates the expulsion of stones but also aids in the repair of the urinary system.

#### 2.2.4 Gut microbiota and urinary calculi

Recent studies highlight the significant role of gut microbiota in the development and progression of kidney stones (13). Dysbiosis in the gut microbiota is commonly observed in patients with kidney stones, with notable differences in the abundance of specific bacterial genera, such as Bacteroides, Prevotella, Lachnoclostridium, Blautia, and Bifidobacterium, compared to healthy individuals (14). A particularly important bacterium, Oxalobacter formigenes, plays a key role in oxalate metabolism. Its reduced abundance is associated with elevated urinary oxalate levels, which increase the risk of calcium oxalate stone formation (15). Probiotic bacteria such as Lactobacillus and Bifidobacterium also contribute to oxalate degradation and lower urinary oxalate excretion (16). Additionally, Bacillus subtilis has been shown to mitigate calcium oxalate stone formation in experimental models (17). Oxalobacter formigenes specifically degrades oxalate in the colon, thereby reducing intestinal oxalate absorption, decreasing urinary oxalate excretion, and lowering the risk of calcium oxalate stone formation (18). A higher baseline abundance of oxalate-degrading genes (oxc and frc) in the gut is associated with enhanced oxalate metabolic capacity, significantly reduced fecal and urinary oxalate levels, and a more favorable therapeutic response to O. formigenes colonization in lowering urinary oxalate (19). Gut microbiota also influences uric acid metabolism. For instance, elevated levels of Prevotella-9 have been linked to increased serum uric acid levels and disturbances in uric acid metabolism, which are closely associated with uric acid stone formation (20). These findings suggest that modulating gut microbiota could serve as a novel strategy for preventing and treating urinary calculi. Approaches such as probiotic supplementation, prebiotic intake, or fecal microbiota transplantation may help restore microbial balance, regulate oxalate and uric acid metabolism, and reduce the risk of stone formation. Furthermore, TCM enema therapy may contribute to gut microbiota regulation, promoting the growth of beneficial bacteria while suppressing harmful ones. This mechanism may partly explain the therapeutic effects of TCM enemas in urinary calculi management.

### 2.3 Clinical applications and efficacy

#### 2.3.1 Clinical application methods

Traditional Chinese medicine (TCM) enemas typically employ the retention enema method. After decocting and concentrating the herbal formula, it is cooled to an appropriate temperature (generally between 37 °C and 39 °C) and slowly introduced into the rectum via a catheter to a depth of approximately 10 to 15 cm. The patient is then advised to maintain a lateral or supine position, retaining the medication for 30 to 60 minutes to ensure adequate absorption. The frequency of enemas is usually one to two times daily, and a treatment course can last 7 to 14 days, depending on the condition (21).

#### 2.3.2 Efficacy evaluation

Clinical efficacy evaluation mainly includes the assessment of stone expulsion, symptom improvement, and changes in renal function indicators. Multiple studies have shown that TCM enema therapy is effective in promoting the expulsion of smaller urinary stones (less than 0.6 cm in diameter) and alleviating stone-related pain. However, for larger or more complex stones, the stone-expelling effect of TCM enema alone may be limited, often necessitating combination with other treatment methods.

## 3 Western medicine enema therapy

### 3.1 Commonly used drugs and their effects

#### 3.1.1 Smooth muscle relaxants

Progesterone is commonly used as a pharmaceutical enema agent (22). As a progestin, it effectively relaxes the smooth muscles of the ureter, with a particularly pronounced effect on the lower segment of the ureter. The mechanism of action involves binding to progesterone receptors on the cell membrane, inhibiting calcium ion influx, and consequently reducing intracellular calcium ion concentration. This leads to the relaxation of smooth muscles, dilation of the ureteral lumen, and a decrease in intraluminal pressure, thereby facilitating the expulsion of calculi. Additionally, progesterone possesses a certain central analgesic effect, which can alleviate symptoms of renal colic. Tamsulosin is another pharmaceutical agent that can be administered via enema. It is a highly selective α_1_ receptor antagonist that selectively blocks α_1_ receptors on ureteral smooth muscles. This action relaxes the smooth muscles of the lower ureter, reducing resistance to ureteral peristalsis and aiding the passage of stones through narrow sections (23). Furthermore, tamsulosin can improve symptoms of bladder outlet obstruction, making it particularly suitable for patients with urinary stones accompanied by lower urinary tract symptoms.

#### 3.1.2 Alkalizing agents

Sodium bicarbonate plays a significant role in the treatment of uric acid stones through pharmaceutical enema. Uric acid stones have low solubility in acidic urine, making them prone to formation and enlargement. Administering sodium bicarbonate via enema can alkalize the urine, increasing the solubility of uric acid and thus promoting the dissolution of uric acid stones. The mechanism involves the absorption of sodium bicarbonate in the intestine, which raises the concentration of bicarbonate ions in the blood. This process decreases the concentration of hydrogen ions in the urine, elevating the pH level and allowing uric acid to dissolve as urate salts, which are then excreted in the urine.

### 3.2 Mechanisms of action

#### 3.2.1 Mechanism of smooth muscle relaxants

Taking progesterone as an example, in addition to binding with progesterone receptors to inhibit calcium ion influx, it also influences intracellular second messenger systems. Specifically, it reduces the degradation of cyclic guanosine monophosphate (cGMP), thereby increasing intracellular cGMP levels. This elevation mediates the relaxation of smooth muscle. Tamsulosin, on the other hand, primarily works by blocking α_1_ receptors, which reduces the contractile effects of norepinephrine on smooth muscle. This action relaxes the ureteral smooth muscle, improving ureteral compliance and coordination of peristalsis.

#### 3.2.2 Mechanism of alkaline medications

The urine-alkalizing effect of sodium bicarbonate is based on the principles of acid-base balance. When the pH of urine increases, the dissociation of uric acid is enhanced, and the solubility of its ionic form, urate, is significantly higher than that of undissociated uric acid molecules. This leads to the gradual dissolution of uric acid stones. Furthermore, an alkaline environment inhibits the formation of uric acid crystals, thereby preventing the recurrence of stones.

### 3.3 Clinical applications and efficacy

#### 3.3.1 Key points of clinical application

In Western medicine enemas, progesterone and tamsulosin can be dissolved in an appropriate amount of saline or injectable water to prepare an enema solution, tailored to the patient’s condition. This is typically administered once or twice daily. Sodium bicarbonate enema solutions are generally prepared at a concentration of 3%. The dosage is adjusted based on the patient’s urine pH, with the enema retained for approximately 30 to 60 minutes, administered once or twice daily over a course of 2 to 3 weeks.

#### 3.3.2 Summary of efficacy

For lower ureteral stones, enema treatment with smooth muscle relaxants has shown some efficacy (24) In treating uric acid stones, sodium bicarbonate enemas, combined with dietary control, can help dissolve smaller uric acid stones or reduce their size. However, for larger stones or those with complex compositions, Western medicine enemas often do not achieve optimal results and are typically used as an adjunct therapy or in combination with other treatment methods.

## 4 Combined TCM and Western medicine enema therapy

The distinct mechanisms and advantages of traditional Chinese medicine (TCM) and Western medicine in treating urolithiasis have garnered increasing attention, particularly in the context of combined enema therapy. For instance, the traditional Chinese medicine Sanjin Paishi Tang is known for its multifaceted effects, such as promoting ureteral peristalsis, regulating urine composition, and providing anti-inflammatory and antioxidant benefits. On the other hand, the Western medicine progesterone effectively relaxes ureteral smooth muscle. When applied together in an enema for patients with urolithiasis, this combination can enhance stone expulsion rates, shorten expulsion time, and alleviate symptoms. A retrospective study involving 190 patients with urinary system stones randomly divided them into a treatment group and a control group, each with 95 cases. The control group received Baishi Qi Tang, while the treatment group received a combination of TCM and Western medicine. The overall efficacy rate in the treatment group was significantly higher than that in the control group (25). Clinical research has found that the stone expulsion rate in the combined treatment group can increase to about 75%, showing a significant advantage over single herbal or Western medicine enema treatments. Despite these promising results, variations in drug compatibility ratios, dosage, and treatment course may influence clinical outcomes. For example, a retrospective study involving 120 patients with urinary system stones randomly assigned them to a treatment group and a control group, each with 60 cases. The control group received combined TCM and Western medicine therapy, while the treatment group additionally used the Da Huang retention enema method. Results indicated that the total efficacy rate of the treatment group was significantly higher than that of the control group. The application of the Da Huang retention enema method in treating urinary system stones can enhance drug absorption, induce defecation response, and promote stone expulsion, demonstrating high clinical application value. The conclusion is that the combination of TCM and Western medicine is effective in treating urinary system stones, relieving pain, increasing urine output, and improving stone expulsion outcomes, thus possessing significant clinical application value. To better illustrate the underlying processes, a schematic diagram summarizing the mechanisms of TCM and Western medicine enema therapies.

## 5 Safety considerations

### 5.1 TCM enema safety

Herbal enemas may pose risks of allergic reactions, particularly for individuals sensitive to proteins, polysaccharides, and other components in certain traditional Chinese medicines. Such allergies can manifest as rashes, itching, and even respiratory difficulties (21). Additionally, the temperature, concentration, and procedural standards of herbal enemas significantly impact their safety. Excessive temperatures may scald the rectal mucosa, while overly concentrated solutions can irritate the intestines, causing discomfort such as abdominal pain and diarrhea. Improper enema administration, such as inserting the rectal tube too deeply or applying excessive force, can lead to severe complications like rectal perforation or bleeding. Furthermore, quality control of herbal medicines is crucial; contamination or the presence of harmful substances like heavy metals in herbal materials can pose health risks to patients.

### 5.2 Western medicine enema safety

Western medicine enemas also present certain safety risks. Long-term use of smooth muscle relaxants such as progesterone may lead to endocrine disorders, with female patients potentially experiencing symptoms like menstrual irregularities and breast tenderness. Tamsulosin can cause adverse reactions such as hypotension and dizziness (26). If alkaline drugs like sodium bicarbonate are administered in excessive doses or if the patient has renal insufficiency, there is a risk of disrupting the body’s acid-base balance, potentially leading to severe consequences such as metabolic alkalosis. Additionally, the procedure of administering Western medicine enemas may result in complications like rectal mucosal damage and infection.

## 6 Future directions

### 6.1 Mechanistic studies

Current research indicates that the integration of traditional Chinese medicine (TCM) with Western medicine shows promising efficacy in the treatment of urolithiasis. However, the precise molecular mechanisms by which TCM exerts its effects remain unclear. Existing studies suggest that TCM may alter urinary pH and osmotic pressure, thereby creating an environment unfavorable for stone formation. Additionally, TCM may enhance ureteral peristalsis, thereby facilitating the expulsion of stones. Future research should delve into the molecular biology and pharmacology of TCM to elucidate its impact on the formation, growth, and expulsion of urinary stones. Techniques such as mass spectrometry could be employed to identify active compounds, while advanced methods like transcriptomics, proteomics, and metabolomics could be used to explore the specific targets of these bioactive substances. Furthermore, combining molecular docking and molecular dynamics simulations could provide insights into how the active components of TCM modulate physiological functions within the urinary system.

### 6.2 Optimizing treatment protocols

Currently, there is a wide variety of herbal enema formulations available, but the lack of standardized protocols remains a significant challenge. Future endeavors should focus on extensive clinical trials and experiments to identify the most effective formulation combinations. For instance, compatibility studies can be conducted on different herbal medicines to discover combinations with synergistic effects, thereby enhancing therapeutic outcomes. Additionally, personalized treatment plans can be developed based on specific patient conditions, such as stone size, location, and composition. For smaller stones, a purely herbal enema treatment might suffice, whereas larger stones might necessitate a combination of Western surgical interventions or other treatment modalities. Enema techniques also require optimization to maximize therapeutic efficacy. The current enema techniques primarily include retention enemas and cleansing enemas. Future research should aim to further refine these methods to increase drug absorption efficiency and therapeutic efficacy. This could involve investigating the effects of various parameters such as enema solution temperature, concentration, and flow rate on treatment outcomes to determine the optimal enema parameters. Additionally, the development of innovative enema equipment and technologies, such as smart enema devices, could enhance the precision and safety of enema administration.

### 6.3 Integration with other medical technologies

Integration with modern medical technologies offers additional opportunities for enhancing treatment outcomes. The use of modern imaging technologies, such as ultrasound, CT, and MRI, facilitates precise diagnosis and localization of urinary system stones. During enema treatment, imaging technologies can be employed to monitor changes in the stones in real-time, allowing for an accurate assessment of therapeutic efficacy. Moreover, multi-omics approaches—including genomics, transcriptomics, proteomics, and metabolomics—can provide comprehensive insights into the pathogenesis of urinary stones. By analyzing patients’ genetic and protein profiles, researchers can identify genes and proteins associated with stone formation, such as specific gene mutations linked to urolithiasis. This knowledge can guide the development of targeted therapies, providing a basis for personalized treatment strategies. Multidisciplinary collaboration further expands the potential of enema therapy. Western medicine can rapidly remove larger stones through surgical procedures or extracorporeal shock wave lithotripsy, while traditional Chinese medicine (TCM) can regulate bodily functions and promote stone expulsion and recurrence prevention through herbal enemas, acupuncture, and massage. Strengthening the integration of TCM and Western medicine in treating urinary stones can leverage the advantages of both approaches for improved therapeutic outcomes. The integration of TCM with Western approaches leverages the strengths of both, enhancing overall therapeutic outcomes. Beyond the integration of TCM and Western medicine, interdisciplinary collaboration with fields such as materials science and engineering can lead to the development of innovative therapeutic devices and materials. For instance, materials with excellent biocompatibility and adsorption properties could be designed to absorb harmful substances within stones, promoting their dissolution and expulsion. Collaboration with engineering could also result in the creation of smart devices for monitoring and treating urinary stones, enhancing the precision and efficiency of treatment.

In summary, the future research directions for enema treatment of urinary stones will become increasingly diverse and in-depth. Through efforts in elucidating the mechanisms of herbal formulations, optimizing treatment protocols, integrating modern medical technologies, and fostering multidisciplinary collaboration, it is anticipated that safer, more effective, and personalized treatment options can be provided for patients with urinary stones. .

## 7 Conclusion

Enema therapy, utilizing both TCM and Western medicine approaches, represents a novel and promising strategy for the treatment of urinary calculi. By leveraging unique mechanisms of action, such as regulating urine composition, enhancing ureteral peristalsis, and modulating gut microbiota, enema therapy offers distinct advantages over traditional treatments. TCM enemas, with their anti-inflammatory and antioxidative properties, complement the smooth muscle relaxant and alkalizing effects of Western medicine enemas, making integrative approaches particularly appealing. However, the clinical application of enema therapy is still in its early stages, and further research is needed to address several challenges. These include optimizing treatment protocols, ensuring safety and quality control, and understanding the long-term effects of enema therapy on stone recurrence and overall urinary health. By addressing these gaps, enema therapy has the potential to become an integral part of the therapeutic arsenal for urinary calculi, offering patients a less invasive, effective, and holistic treatment option.
